# Vertically-Aligned Multi-Walled Carbon Nano Tube Pillars with Various Diameters under Compression: Pristine and NbTiN Coated

**DOI:** 10.3390/nano10061189

**Published:** 2020-06-18

**Authors:** Amir Mirza Gheitaghy, René H. Poelma, Leandro Sacco, Sten Vollebregt, Guo Qi Zhang

**Affiliations:** Department of Microelectronics, Delft University of Technology, Feldmannweg 17, 2628CT Delft, The Netherlands; R.H.Poelma@tudelft.nl (R.H.P.); L.N.Sacco@tudelft.nl (L.S.); s.vollebregt@tudelft.nl (S.V.); G.Q.Zhang@tudelft.nl (G.Q.Z.)

**Keywords:** carbon nanotubes, conformal coating, NbTiN, nano-indentation, LPCVD

## Abstract

In this paper, the compressive stress of pristine and coated vertically-aligned (VA) multi-walled (MW) carbon nanotube (CNT) pillars were investigated using flat-punch nano-indentation. VA-MWCNT pillars of various diameters (30–150 µm) grown by low-pressure chemical vapor deposition on silicon wafer. A conformal brittle coating of niobium-titanium-nitride with high superconductivity temperature was deposited on the VA-MWCNT pillars using atomic layer deposition. The coating together with the pillars could form a superconductive vertical interconnect. The indentation tests showed foam-like behavior of pristine CNTs and ceramic-like fracture of conformal coated CNTs. The compressive strength and the elastic modulus for pristine CNTs could be divided into three regimes of linear elastic, oscillatory plateau, and exponential densification. The elastic modulus of pristine CNTs increased for a smaller pillar diameter. The response of the coated VA-MWCNTs depended on the diffusion depth of the coating in the pillar and their elastic modulus increased with pillar diameter due to the higher sidewall area. Tuning the material properties by conformal coating on various diameter pillars enhanced the mechanical performance and the vertical interconnect access (via) reliability. The results could be useful for quantum computing applications that require high-density superconducting vertical interconnects and reliable operation at reduced temperatures.

## 1. Introduction

Carbon nanotube (CNT) based structures have attracted widespread scientific interest due to their exceptional mechanical and electrical performance rendering them as promising candidates for advanced applications on various scales [1]. On the macroscopic scale, great progress has been made in CNT actuations by coupling their mechanical and electrical properties in electrostatic [2], electrochemical [3], and electro-thermal [4] actuations. On the micro/nanoscopic scale, CNTs have demonstrated promising applications in Nano Electro Mechanical Systems (NEMS) through successful demonstrations on high-frequency oscillators [5], rotational actuators [6], nanometer tweezers [7], drug delivery [8], and vertical interconnect access (via) [9]. Extremely attractive performances of individual tubes are difficult to reach when they are assembled in the large bundles necessary to make real vias or lines in integrated chips. Two proposed approaches to overcome the current limitations are either to make very tiny local connections that will be needed in future advanced chips or to make carbon–metal composite structures that will be compatible with existing microelectronic processes.

CNTs for electrical interconnects application in integrated chips (IC) have been studied since 2001 [10], but few researchers have investigated CNTs as superconductor interconnect applications and specifically the mechanical strength of the structure under compression for practical applications. One of the required functions for superconductive via is operating under the mechanical loads of packaging. Using the CNT pillar as through silicon via (TSV) filling material [11] or as microbumps in flip-chip packaging [12], needs mechanical strength under compression. To make the superconductive vias, a thin layer of superconducting material with a thickness larger than the London penetration depth (50 nm) is needed to effectively shield radiation [13]. The usual method for superconductive VIA is to deposit a thin layer of superconductive material inside the TSV [14] or to use superconductive microbumps [15].

CNTs are the strongest and stiffest materials in terms of tensile strength, but not nearly as strong under compression. Because of their hollow structure and high aspect ratio, individual tubes tend to undergo buckling when placed under compressive, torsional, or bending stress [16]. Compressive performance of various pristine CNT forms such as individual [17], forest [18], film [19], turf [20], array [21,22], and pillars [23], have been considered in the literature. Among them, vertically aligned (VA) CNT pillars have been recognized as a promising structural material for the fabrication of high aspect ratio (AR) 3D micro- and nano-architectures [24,25]. VACNT pillars represent a promising class of mechanically strong and resilient lightweight materials, capable of supporting large reversible deformation and absorbing mechanical energy [26]. The mechanical response of VACNTs to uniaxial compression depends on various factors, including the material microstructure, density, height, rate of deformation, and the nature of the interaction between the CNTs and the compressing indenter [27]. For instance, vertical gradients in CNT morphology dictate the location of incipient buckling and distinct deformation mechanisms [18]. In addition, the horizontal gradient along the direction of the precursor flow varies bulk density and affects the peak stress and the modulus of the freestanding CNT arrays [28]. In order to tune the compressive strength of CNTs, various methods of mixing with epoxy composite [29], processing with CVD treatment [30], and coating with hard materials [31] have been investigated.

Beside the investigation of pristine CNTs, researchers have made a considerable effort to optimize the full potential of individual CNTs by application of conformal coatings of metal [32], ceramic [33] or even graphene [34]. Coated CNT arrays have received significant attention for potential use in electrical [35], optical [36], thermal [37], chemical [38], and mechanical [31] fields. Though the compressive performance of coated CNTs is expected to contribute significantly to the performance of these applications, relatively little is known about the collective mechanical behavior of coated CNT arrays and how their morphology may influence the mechanical response. Abadi et al. [39] investigated the mechanical properties of CNT forests coated with alumina using an atomic layer deposition (ALD) process. Poelma et al. [40] investigated the compressive strength of CNT arrays coated with SiC using the low-pressure chemical vapor deposition (LPCVD) process. In both publications, the material properties could be accurately tuned using different coating thicknesses and the compressive strength increased as a function of the coating thickness.

Recently the use of coated CNT as an electrical superconductor was presented. Kim et al. [41] fabricated superconducting fiber by sputter deposition of an NbN layer on free-standing CNT sheets, followed by post-twisting. Salvato et al. [42] proposed using Nb deposited on MWCNT film as a horizontal interconnect in superconducting nanoelectronics. The coating approach offers an interesting solution to fabricate fine pitch, high aspect ratio (AR), and superconductive interconnect by conformal superconductive coating to vertically aligned CNT pillars. The CNT bundle can provide sufficient toughness and high AR matrix, and meanwhile, the superconductive coatings do not only provide superconductivity, but can also improve the morphology and density of the CNT pillar, and therefore the mechanical properties of the pillar.

In this study, we first fabricated various diameter (30–150 µm) MWCNT pillars with a height of 120 µm by catalytic LPCVD. Then, the conformal coating of VA-MWCNT pillars was performed by ALD, with 20 nm thickness of niobium-titanium-nitride (NbTiN) as superconductor material. The morphology and structure of pristine and coated VA-MWCNT were investigated with scanning electron microscopy (SEM), transmission electron microscopy (TEM), focused ion beam (FIB) and energy-dispersive X-ray spectroscopy (EDX). To characterize the compressive response, flat-punch nano-indentation measurements on pristine and coated CNT pillars were performed. We analyzed the mechanical response by measuring the force-displacement curve and SEM inspection after compression. The proposed structure opens new perspectives on the possibility of using VA-MWCNT pillar as vertical interconnect for superconducting applications.

## 2. Materials and Methods

### 2.1. VA-MWCNT Pillar Growth with LPCVD

Growth of CNT on a silicon wafer needs three main ingredients: a barrier layer, catalyst nanoparticles, and a carbon source. The barrier layer prevents the diffusion of the catalyst into the silicon substrate and enables the transition metal catalyst film to break up into nanoparticles instead of forming an agglomeration. Regarding superconductor interconnect applications, an electrically conductive layer has to be used. Particularly, TiN is a diffusion barrier often used in semiconductor technology, which has the advantage of being electrically conductive at room temperature and can be superconductive at 4K. A transition metal catalyst which can be used on TiN to form CNTs is Fe, as was shown in previous work [43]. Fe nanoparticles are among the most used catalysts due to their high surface energy. However, it is important to point out that their size, structure, composition, type, and state of catalyst all influence CNT growth. In this study, LPCVD was employed to grow vertically aligned CNTs due to its scalability and the ability to grow long, high-density CNTs on controllable locations by patterning the catalyst.

**Figure 1:**
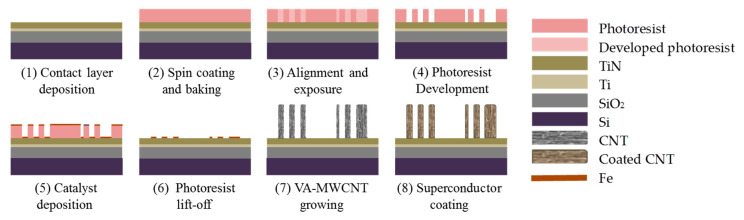
Schematic of microfabrication process; (1) SiO_2_ deposited and Ti/TiN (10/50 nm) sputtered on a silicon wafer as a barrier layer, (2) photoresist (1.4 µm) coated and baked, (3) wafer aligned and exposed under the photomask, (4) pattern developed, (5) Fe nanoparticle (5 nm thickness) evaporated as a catalyst, followed by, (6) a lift-off process to define the CNT growth regions, (7) Vertically aligned multi-walled carbon nanotube (MWCNT) bundles (120 µm) grown in an AIXTRON Black Magic chemical vapor deposition reactor, (8) Finally, NbTiN (20 nm) deposited by atomic layer deposition (ALD) on the structure.

Figure 1 represents the fabrication process of the coated VA-MWCNT pillars. We use a 100 mm p-type (100) silicon wafer as a substrate for CNT growth [44]. First, a 500 nm thick thermal silicon oxide layer is grown for electrical insulation of the TiN from the substrate. Next, a 10/50 nm layer of Ti/TiN is sputtered to enable the CNT growth from the catalyst particles. Then, a 5 nm thin layer of iron (Fe) catalyst is deposited by electron beam evaporation. The catalyst is patterned using optical lithography and a lift-off process. For the lift-off process, we spin coat a film of 1.5 µm thick negative photo-resist (AZ Nlof2020, Microchemicals GmbH) and use an NMP solvent at 70 °C for dissolving the resist during the lift-off. Next, MWCNT pillars are grown by LPCVD in a commercial deposition system (Black Magic Pro, Aixtron, Herzogenrath, Germany). The CNTs are grown at a temperature of 650 °C using a gas flow mixture of 700 sccm hydrogen over 50 sccm acetylene (H_2_/C_2_H_2_) at 80 mbar for 10 min.

### 2.2. NbTiN Coating on CNT Pillar with ALD

NbTiN is widely applied in superconducting electronic devices due to its merits of having a wide energy gap, high superconducting temperature, and mitigated requirement as well as compatibility with microfabrication processes [45]. For uniform deposition on high aspect ratio CNTs, atomic layer deposition (ALD) was used because it provides excellent homogeneity, high surface conformity, and low pinhole density along with nearly perfect thickness control.

NbTiN with a thickness of 20 nm was deposited on VA-CNT pillar with an Ultratech/CNT Fiji ALD system. Tris(diethylamido)(tert-butylimido) niobium (TBTDEN) and tetrakis(dimethylamino) titanium (TDMAT) were used as the niobium and titanium precursors and were maintained at 100 °C and 75 °C, respectively. The precursors were delivered to the system with a Boost^TM^ system which introduces a charge of argon gas into the precursor cylinder before pulsing the precursor into the ALD reactor and substantially improving the transfer of low vapor pressure material from the cylinder to the substrate surface [46]. The ALD process consisted of repeated supercycles of precursor pulses and plasma exposure alternated with fixed purge periods. An individual Nb sequence consisted of a 500 ms boosted pulse of TBTDEN followed by a 5 s purge, repeated 3 times. An individual Ti sequence consisted of a 750 ms pulse of TDMAT followed by a 5 s purge. The plasma exposure times for the Nb and Ti sequences were 40 s and 20 s, respectively. For Ti cycles, the plasma gas contained 5 sccm N_2_, while an additional 80 sccm H_2_ was delivered during the Nb cycles. The plasma power was kept at 300 W for ALD runs. The layer thickness was controlled by the number of cycle/timing of the deposition process for 122 cycles, with a deposition rate of about 0.5 Å/min.

### 2.3. Compression Testing with Nano-Indentation

Nano-indentation can be used for testing the mechanical properties of microscale materials. The method employs high precision instrumentation to monitor the force and displacement of an indenter tip during compressive loading of a sample [47]. To evaluate the elastic properties as a continuous function of penetration depth, the continuous stiffness measurement (CSM) technique introduces relatively high-frequency loading and unloading cycles by imparting a small sinusoidal displacement at the indenter tip superimposed upon the larger steady tip displacement rate [48]. In such a way, stiffness and elastic properties may be evaluated at each data collection interval. The measurement of critical strain and average modulus is determined according to the classical three-region stress–strain curves, which have been reported widely in polymer and foam/cell material compression [49].

In this research, Nano-indentation was used to understand the effect of brittle coating on the mechanical performance of superconductive interconnects which is vital to the reliability of microsystem packages. The effect of coating on the compressive response of CNT pillars was characterized using an Agilent MTS nano-indenter XP G200. The Nano Indenter G200 is powered by electromagnetic transducers and utilizes the XP indentation head with a loading capability of 500 mN, delivering 500μm maximum indentation depth and 10 pm displacement resolution. Vertically oriented cylindrical pillars with an average height of about (120 ± 5) µm and diameters ranging from (30 ± 1) µm to (150 ± 2) µm were synthesized for cyclic compression tests. The uniaxial compression test of the CNT pillars was performed in air using a diamond punch tip of 150 μm diameter. The flat surface of the tip enables accurate detection of the CNT pillar surface and maintains a uniform contact area during compression. The pillars were compressed for 70% of strain and the compressive stress–strain response was recorded only for the loading process. The CSM parameters used were 2 nm amplitude, 45 Hz frequency, sensitive 100 N.m^−1^ surface detection and a strain rate of 0.01 s^−1^.

### 2.4. Pillar Characterization

The microstructure and morphology of the pristine and 20 nm-coated VACNT along the longitudinal direction were characterized by SEM (FEI XL50, Hillsboro, OR, USA). TEM and elemental mapping were measured using an FEI Titan. For the TEM analyses of all the synthesized samples, cross-section lamella preparation was prepared within a Scios DualBeam instrument, a combination of an SEM operating at 30 kV that produces enlarged images of the studied materials and an FIB system containing a Gallium(Ga) source that enables fast and precise milling of the materials [50]. The average CNT diameters and number of layers were obtained with over 10 individual multi-walled carbon nanotubes (MWCNTs) in different TEM images. The cross-section area and second moment of the area were deduced from the average outer/inner diameters of the nanotubes.

The chemical inertness of CNT requires some degree of defect for controlled deposition through ALD. The type, quantity, and distribution of such defects rules the deposition rate and defines the growth behavior. Raman spectroscopy was used to gather further information on the defect degree of the VACNTs. We conducted Raman spectroscopy measurements on three VACNT samples of each type at five different characterization positions along the longitudinal direction from the top to the bottom of each sample. We used the I_G_/I_D_ ratio to correlate the structural purity of the graphitic materials to the disordered graphite via X-ray diffraction. Raman characterization was done using a Renishaw inVia system with a 514 nm wavelength Ar+ laser to determine the presence of defects on the CNTs and the effects of NbTiN deposition.

## 3. Results

### 3.1. Morphological and Structural Characterization

#### 3.1.1. SEM and TEM Images

**Figure 2:**
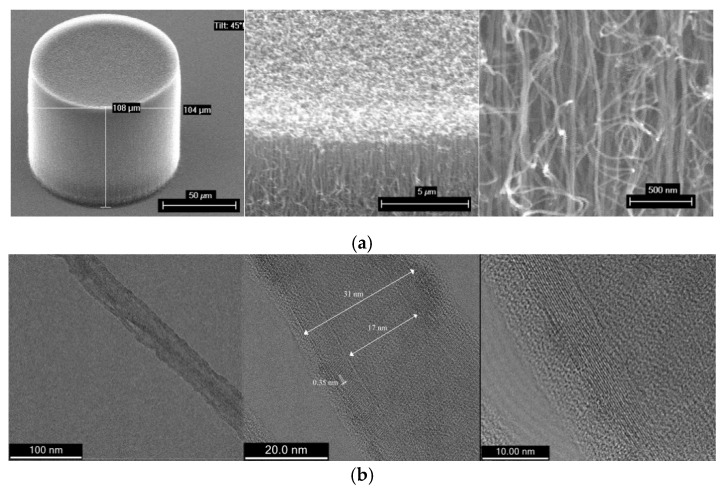
(**a**) Scanning electron microscopy (SEM) images of pristine vertically aligned (VA)-MWCNT pillar and (**b**) transmission electron microscopy (TEM) images of pristine MWCNT, at different magnifications.

The surface morphology of the VACNTs pillars and their CNTs structure were examined by SEM and TEM. Figure 2 summarizes the as-grown CNT pillar with circular cross-sections and their nanostructures. Figure 2a shows SEM images that qualitatively illustrate the aligned distribution and morphology of the VACNTs along the longitudinal CNT direction. Figure 2b shows representative TEM images of MWCNTs extracted from the VA-MWCNT pillar. From the ensemble of these images, it was calculated that the nanotube average diameter, is 30 ± 4 nm and the tube density within the pillar is around 110 ± 10 tubes/µm^2^ with homogeneity, high surface conformity, and low pinhole density along with nearly perfect thickness control.

**Figure 3:**
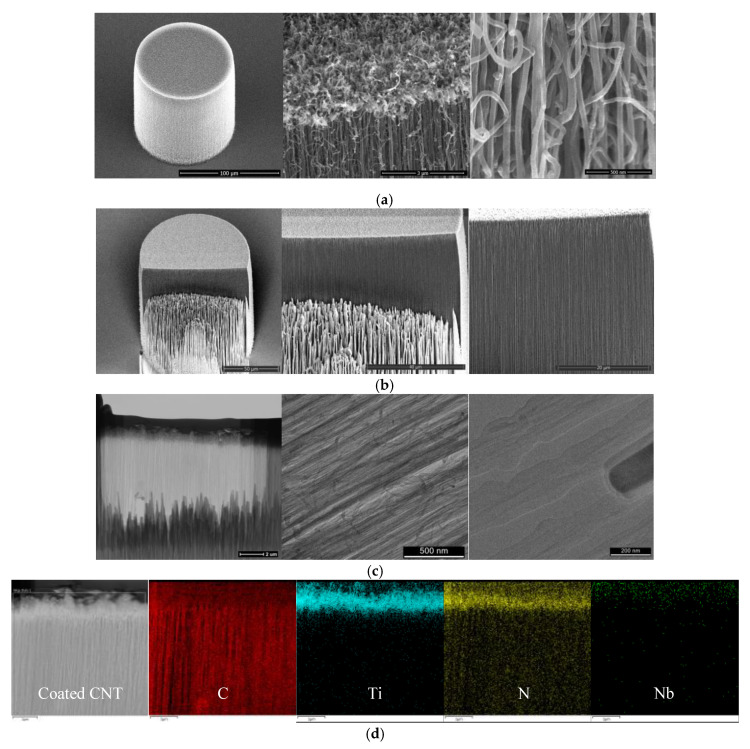
VA-MWCNT pillar with 20 nm NbTiN coated. (**a**) SEM images of coated VA-MWCNT pillar; (**b**) SEM image of FIB on pillar; (**c**) STEM and TEM images of focused ion beam (FIB) prepared lamella; (**d**) energy-dispersive X-ray spectroscopy (EDX) mapping of the lamella.

Figure 3 shows the morphology analysis of the MWCNT pillar after 20 nm NbTiN coating. Figure 3a shows the SEM images at different magnifications which reflect uniform deposition around the nanotube at top and bottom. The initial vertical orientation and the high surface area of the CNT array are retained, while in correspondence to the CNT–CNT junctions some localized aggregations are formed. The average coated CNT diameter is 60 ± 7 nm. Therefore, the average coating thicknesses deposited on the MWCNT surface is about 18 nm. To investigate the morphology and the coating infiltration depth within the VA-CNT arrays, we performed FIB preparation in Figure 3b with further analysis by TEM, as shown in Figure 3c. Due to changing the coating structure by using high energy ion milling, we also scratched the pillar with the needle to estimate the penetration of coating. The coating uniformity and the penetration depth was investigated along the radial and vertical direction. Cycle-to-cycle variations in both precursor dose and purging time were used to achieve a uniform monolayer on a CNT [51]. The coating thickness reduces roughly 2 nm for every 1 µm from the sidewall to the core. The penetration depth from the top is less than 5 µm. The low penetration depth and the uneven uniformity of the ALD coating within the intricate CNT foam-like morphology has been the object of several studies [52,53]. Figure 3d shows the emission profiles of carbon, titanium, nitrogen, and niobium. The Ti, N, and Nb elements are relatively homogeneously concentrated on top of the pillar, indicating a low diffusion of coating inside the dense pillar, which agrees well with the FIB results.

#### 3.1.2. Raman Spectroscopy

**Figure 4:**
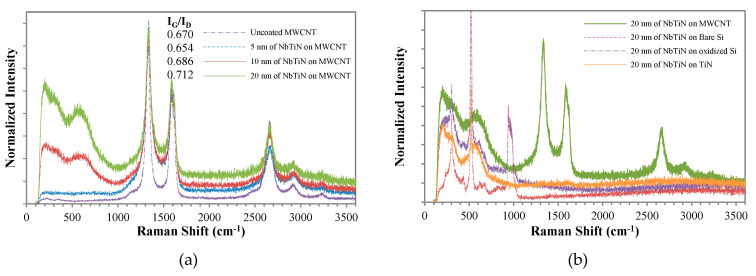
Normalized Raman spectra of (**a**) coated MWCNT with different thicknesses of NbTiN and (**b**) 20 nm NbTiN on various points of the substrate.

Figure 4a shows the Raman spectrum intensity of the CNT pillar which was normalized to the G-peak intensity. The peaks near 1350 cm^−1^ and 1580 cm^−1^ in the first-order region correspond with the defects of any kind (D) and sp^2^ carbon (G) modes of the CNTs. The G-peak has convolved with a shoulder peak at 1620 cm^−1^, which is known as the D’-peak and is also associated with defects within the graphite hexagons originating from different points in the Brillouin zone. The intensity of the disordered graphite peak, with respect to the G-peak, refers to the number of defects present in the graphitic shells. The intensity ratio I_G_/I_D_ of the peaks can be used to evaluate the quality of the CNTs, a higher ratio generally indicates better quality.

The data shows modulation of the graphite (G) and disordered graphite (D) peaks due to the broad NbTiN Raman features at lower wavenumbers, as is also visible in the spectrum of NbTiN on TiN in Figure 4b. For thicker NbTiN coatings the noise in the spectrum also increases, as the signal from the CNT is reduced. Deconvolution of the peaks using a least-square fitting procedure showed that the intensity ratio I_G_/I_D_ is equal to 0.67 for uncoated tubes, revealing the low crystallinity degree of the CNT sample. Therefore, due to their intrinsic defectivity, no functionalization treatments were performed on the CNT scaffold before the coating procedures as defects can act as absorption sites. No relevant changes of the spectra of the CNTs occurred during the ALD deposition, suggesting that the CNTs were not damaged by the coating process, the apparent increase in I_G_/I_D_ (which suggests an improvement of crystallinity) is attributed to inaccuracies in the fitting.

Figure 4b shows the Raman spectra from various points on the substrate of a 20 nm NbTiN layer. Deposition of NbTiN directly on bare and oxidized Si substrate shows a sharp feature at 520 cm^−1^ and a smaller feature around 970 cm^−1^ which originate from the crystalline Si substrate. Besides that several broad features are visible below 1000 cm^−1^ which likely originate from the NbTiN.

### 3.2. Mechanical Characterization

#### 3.2.1. Stiffness of VA-MWCNT with Various Diameters

**Figure 5:**
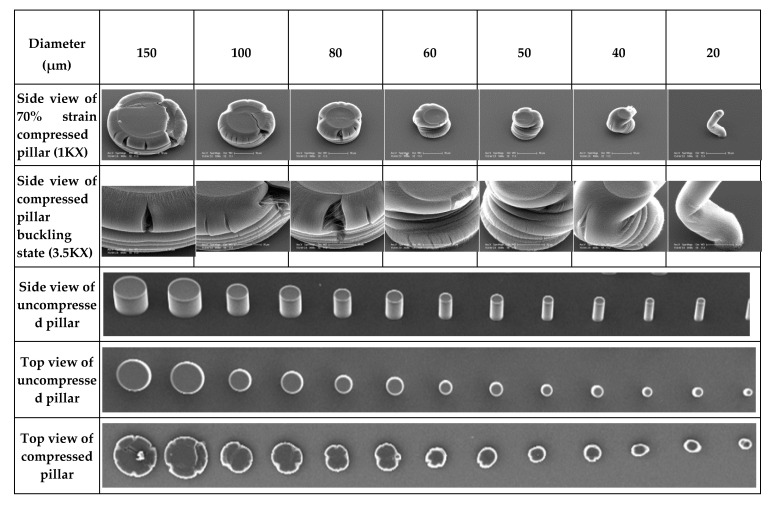
SEM images of pristine CNT pillars after 70% compressed strain of various diameters.

Indentation testing was performed on CNT pillars with the same height and various diameters. SEM images of pristine CNT pillars after 70% compression, as shown in Figure 5, reveal that the pillar failure mode is a type of localized periodic buckling. The deformation was similar to the deformation of uncoated CNT pillars in a prior study [40]; the buckling moves spatially along the pillar axis, which initiates at the base and propagates upwards throughout the entire bundle for increased compression depth. Large diameter pillars with an aspect ratio (AR) about 1 (D ~ 80–150 μm) show buckles at wavelength of about 2–5 μm and the smaller diameter pillars with AR of 0.5 (D ~ 40–60 μm) show buckling in the wavelength range of 10 to 15 μm. For needle-like pillars with AR of less than 0.25 (D ~ 20–30 μm), the bending occurs from the middle of the pillar. These typical buckling characteristics appear to be unique for CNT pillars. More importantly, the localized periodic buckling events are very reproducible and in excellent agreement with the in-situ CNT pillar compression observations from Hutchens et al. [54]. Smaller pillars tend to recover most of their original height upon release of a compressive load, allowing them to be repeatedly loaded.

The criterion for ductile behavior according to classical continuum mechanics cannot be directly satisfied for pristine VA-MWCNT arrays. Collective bending of the individual CNTs inside the discrete structure of the pristine array during compression causes interacts which stick to each other through van der Waals forces. During unloading, the CNTs remain stuck to each other thus preventing full elastic recovery. This localized bending and stiction-like deformation of the CNTs inside the pillar can be perceived as a permanent deformation of the pillar which can exhibit as high ductility.

**Figure 6:**
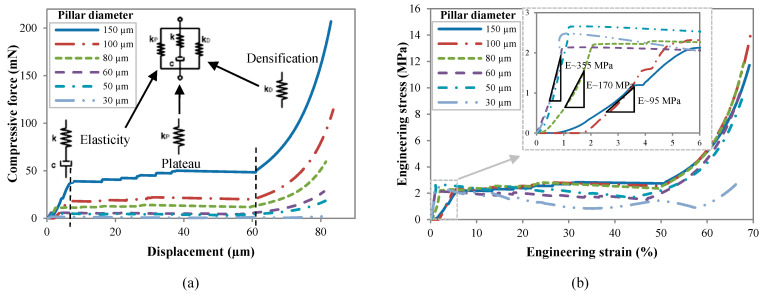
(**a**) Compression force-displacement and (**b**) stress–strain curves for pristine CNT pillars of various diameters.

The compressive load-displacement and stress–strain response of uncoated CNT pillars are shown in Figure 6a,b, respectively. Engineering stress and strain were calculated using the initial diameter and height of the pillar [55]. Despite their complex hierarchical microstructure, the general compressive behavior of VACNTs is akin to that of typical open-cell foams, as was first reported by Cao et al. [56]. In the spirit of the overall foam-like response, the stress–strain curves of VACNTs are characterized by three distinct regimes: (1) a short initial linear elastic section (<5%) up to the yield point followed by (2) a sloped oscillatory plateau (5–50%) with characteristic wavy features corresponding to buckle formation and (3) subsequent hardening to densification (>50%) characterized by rapid stress increase and finally locking. At the scale of its constituents, however, the response of VACNTs is quite different from that of traditional foams. In VACNT bundles, the post-elastic compressive strain is accommodated entirely via the formation of lateral folds or buckles usually close to the bottom of the bundle, while the remaining portion remains virtually unscathed. This is in contrast to traditional foams, where cell-edge bending and cell collapse are primarily responsible for the elastic-plastic foam response. In our VACNT samples, the formation of the first buckle at the bottom signals the transition from elastic to plateau regime. The inherent axial property gradient is responsible for the sequential nature of the buckling. Each buckle is of the order of 10–20 μm in size (depending on pillar diameter) so that several tens of buckles form during deformation. The post-elastic plateau region in the stress–strain curve also shows marked differences from that of an open-cell foam response, as evidenced by its nonzero positive slope and wave-like shape, where each undulation can be traced to subsequent buckling events. Unstable deformation and localization showing plateaus and serrations is highly stochastic, as the stress–strain curves are manifested by multiple strain bursts [57] and the size-dependent serrated behavior can be interpreted through combined gradient-stochastic models [58].

#### 3.2.2. Stiffness of 20 nm Coated VA-MWCNT with Various Diameters

**Figure 7:**
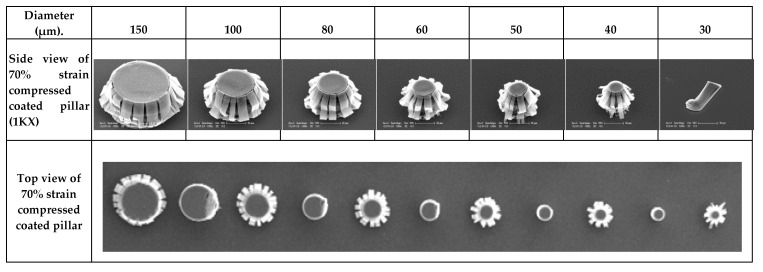
SEM images of 20 nm NbTiN coated CNT pillars after 70% compressed strain of various diameters.

**Figure 8:**
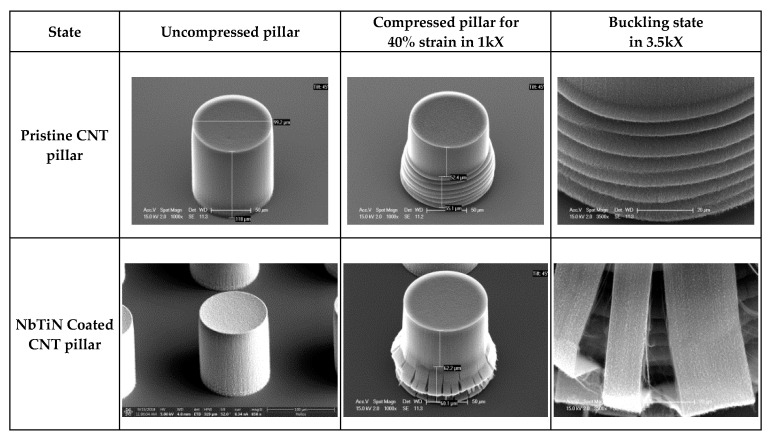
SEM images of compressed coated and pristine CNT pillars with 100 μm diameters after 40 μm compression.

Figure 7 shows the deformation of the CNT pillar coated by the ALD method. For all pillar diameters, for the NbTiN that coats the pillars the brittle fails, and the MWCNTs exhibited formation of local periodic buckles starting from the bottom of the CNT pillar, as evidenced in Figure 8, where coated and pristine pillars are shown. It was concluded that NbTiN only penetrates inside the pillar for a few micrometers as already characterized. Therefore, the MWCNTs at the surfaces of the pillar are coated instead with internal MWCNTs in the pillar. This coating mode explains that CNTs buckle at the top of the buckled nanotubes and gradually develop upwards with applied loading. In this way, the buckling waves progressively develop upwards one by one with strain increase.

As shown in Figure 8, for coated and pristine CNTs as an example of the nanotube buckling process, the bottom of the nanotubes have already buckled and deformed, while the top of the nanotubes have started to buckle progressively. The plastic deformation of pristine CNTs is linearly dependent on strain and reaches up to 40% when the strain is 60%; while the coated CNTs have less than 30% plastic deformation in the same strain range. Theoretically, the larger bending stiffness of individual CNTs within the arrays could lead to better recovery effects and resilience. Localized damage of the coating material in foams causes softening in concentrated regions and leads to fluctuations on the stress plateau in its macroscopic behavior. Hybrid foam with high-ductility coating will have a failure mode similar to that exhibited by homogeneous metal foams. Otherwise, hybrid foams with low-ductility coatings will exhibit a more brittle failure and non-uniform damage regions; especially highly reinforced foams through thicker or stronger coatings.

**Figure 9:**
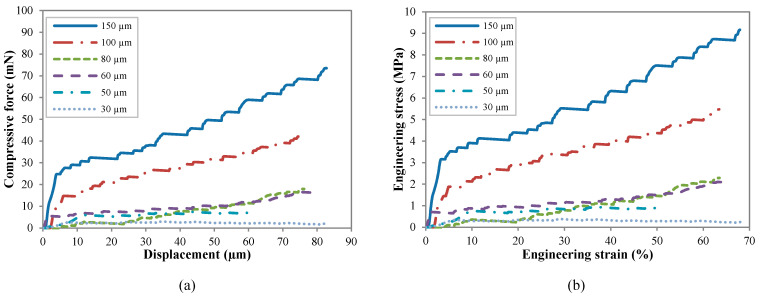
(**a**) Compression force-displacement and (**b**) stress–strain curves for 20 nm NbTiN coated CNT in various diameters.

Figure 9 reveals that the larger CNT pillars show the highest elastic modulus, suggesting that an area of coated CNT structure may play an important role in large strain compression when nanotubes start to contact each other and the behavior of CNTs changes from buckling to packing and folding. Plateau stress increases stepwise with some variation corresponding to the formation and propagation of localized strain bands [59]. It seems that the coating prevents the densification phase under compression. In comparison to pristine pillars, the compressive stress magnitude of larger coated CNTs is more due to the area of the coating. For smaller pillars (D < 60 µm) the stress magnitude of the coated pillar is less than a pristine pillar. Besides, the plateau regime in coated pillars increases with more stable steps in magnitude in comparison to pristine CNTs.

### 3.3. Modelling

Despite the difference in local response of the foam with CNT, it is possible to use the foam-like model to predict the behavior of CNT pillar with highly porous nature. In CNTs, the accommodation of strain during uniaxial compression is accomplished entirely through the formation of folds or buckles of small regions of the structure while the remaining portion remains nearly undeformed. This superposition of an overall foam-like response with localized strain accommodation is the key characteristic of CNT deformation [60].

**Figure 10:**
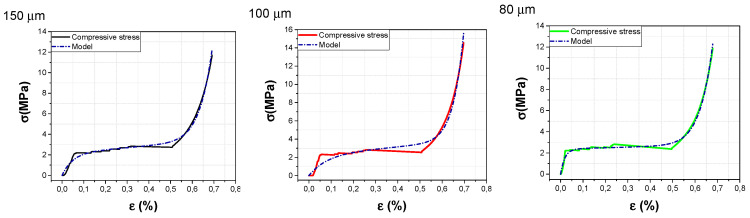

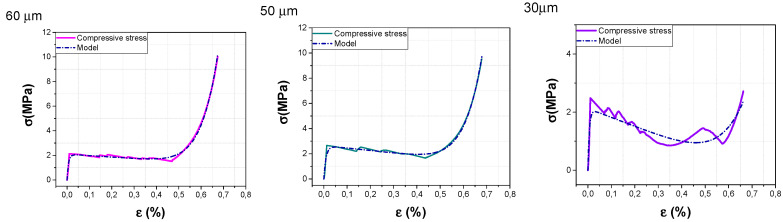
Measured and fitting curves for stress–strain curves for pristine CNT pillars with diameters ranging from 150 µm to 30 µm.

Stress–strain experimental data were fitted to Equation (A8) in Appendix A using MATLAB and the build-in curve fitting toolbox. The extracted parameters for pristine MWCNT are presented in Table 1. Figure 10 shows good agreement between the curve fitting of the foam models with the experimental tests from pristine pillars with diameters ranging from 30 µm to 150 µm. Even for 30 µm pillars with no plateau region which is attributed to the different bending behaviors, we observed a good match. By increasing the diameter of the pillars, the Young’s modulus generally decreased which might be attributed to better alignment and a lower level of waviness along the length for smaller diameters. From a material point of view, it is interesting to specify the relaxation time which is given by the ratio of the damping coefficient C to the spring coefficient K of the Maxwell arm. Relaxation time is a characteristic time length that indicates the amount of viscous forces of the Maxwell arm with respect to elastic forces with unit of time. In Table 1, extracted relation times (T) revealed that pillars with a 100 µm diameter have the highest viscoelastic behavior, which drastically decreases for pillars ranging from 80 µm to 30 µm. Plateau regime represents the bulk of energy absorption in the material, since the area under this region of the stress–strain curve corresponds to the work done on the material. Regarding the plateau region, CNT pillars of 150 µm and 100 µm have similar positive values, revealing the fact that the stress is higher than the damper viscosity, which is not the case for CNT pillars with lower diameters. The intensification region is characterized by a strong non-linear behavior with an exponential function, which is determined by the parameters γ and n of the proposed model. The γ values are similar for all CNT pillars diameters, except for pillars with 30 µm diameter. This can be attributed to the fact that the intensification regime is not fully reached in this case.

The future directions of the present research must include experimentation of in situ and operando SEM nanoindentation observations to consider the complex deformation mechanisms involved in the VACNT pillar arrays. These can shed light on array deformation and the permanent deformation through damage mechanisms in order to obtain a better understanding of the transitions among the three compression regimes which can be incorporated in a future model.

## 4. Conclusions

This paper presented the nano-indentation test on a coated VA-MWCNT pillar for superconductor applications. The compressive stress–strain measurements of pristine VA-MWCNT showed that the three-regions of elasticity, plateau, and densification regime were nearly independent of pillar diameter. The curve fit modelling for foam-like behavior was used to predict the behavior of the CNT pillar. The coated pillars showed step-increase of the stress magnitude due to the brittle coating around the pillar which depended on the coverage area for various diameters. The aim was to assess the mechanical response and fracture mechanism of a brittle coated VA-MWCNT pillar as a vertical superconductor interconnect. The results showed that characterizing and understanding material behavior is important for designing new systems.

## Figures and Tables

**Table 1:** Model parameters for foam-like behavior of pristine vertically-aligned multi-walled carbon nanotube (VA-MWCNT) pillars.

Pillar Diameter [µm]	K [MPa]	C [MPa·Sec]	T = C/K [Sec]	k_P_ [MPa]	γ [MPa]	*n*
150	43 ± 0.5	215 ± 5	5.0 ± 0.2	1.8 ± 0.1	13.1 ± 0.1	8
100	30 ± 1	226 ± 12	7.5 ± 0.6	2.2 ± 0.2	16.0 ± 0.1	10
80	108 ± 3	270 ± 3	2.5 ± 0.1	−1.30 ± 0.07	17.5 ± 0.5	6
60	307 ± 18	209 ± 3	0.70 ± 0.05	−1.10 ± 0.06	16.5 ± 0.1	6
50	270 ± 11	185 ± 6	0.70 ± 0.05	−1.90 ± 0.06	14.0 ± 0.6	6
30	344 ± 50	212 ± 4	0.6 ± 0.1	−3.00 ± 0.15	4.4 ± 0.2	4

## Appendix A

The foam model consists of three systems in parallel (Figure 6a). The first, referred to as the Maxwell arm, contains a spring (stiffness *k*) and dashpot (viscosity *c*) in series. The other two systems contain only springs (*k_P_* and *k_D_*). This model can be used to accurately predict the general shape of the compressive stress–strain curve. Stiffness coefficient *k* is equivalent to the foam Young’s modulus, damping coefficient *C* is equivalent to plateau stress, stiffness *k_P_* represents the slope of the plateau stress and stiffness coefficient *k_D_* is a function of strain.

To model this system, the following physical relations for parallel and series components must be realized [61]. The compressive stress (*σ*) and strain (*ε*) satisfy:

(A1a) $$\sigma_{M}=\;\sigma_{K}=\sigma_{C}$$

(A1b) $$\varepsilon_{M}=\varepsilon_{C}+\varepsilon_{K}$$

(A1c) $$\sigma =\sigma_{M}+\sigma_{P}+\sigma_{D}$$

(A1d) $$\varepsilon =\varepsilon_{M}=\varepsilon_{P}=\varepsilon_{D}$$

σ and ε are related as:

(A2a) $$\sigma_{K}=k\varepsilon_{K}$$

(A2b) $$\sigma_{C}=c\;\frac{d\varepsilon_{C}}{dt}$$

(A2c) $$\sigma_{P}=k_{P}\varepsilon_{P}\;$$

(A2d) $$\sigma_{D}=k_{D}\varepsilon_{D}=\gamma {\left(1-e^{\varepsilon_{D}}\right)}^{n}\varepsilon_{D}\;$$

where $k_{D}$ is 2-parameters exponential function of strain.

(A3) $$k_{D}\left(\varepsilon \right)=\gamma {\left(1-e^{\varepsilon }\right)}^{n}$$

Then, the time evolution is governed by:

(A4) $$\frac{d\varepsilon_{M}}{dt}=\;\frac{d\left(\varepsilon_{C}+\;\varepsilon_{K}\right)}{dt}=\frac{1}{K}\frac{d\sigma_{M}}{dt}+\frac{1}{C}\frac{d\sigma_{C}}{dt}=\;\frac{1}{K}\frac{d\sigma_{M}}{dt}+\frac{1}{C}\sigma_{M}$$

During our experiment strain rate was 0.01 s^−1^. Then, *σ*(*t*) satisfies:

(A5) $$\sigma_{M}\left(t\right)=e^{\frac{-Kt}{C}}\left(e^{\frac{Kt}{C}}-1\right)C\frac{d\varepsilon_{M}}{dt}$$

Applying a substitution of time t by strain $\varepsilon_{M}$, gives:

(A6) $$t=\;\frac{\varepsilon_{M}}{\frac{d\varepsilon_{C}}{dt}}\;\mathrm{and}\;\frac{d\varepsilon_{C}}{dt}=0.01\;s^{-1}$$

and substitution in Equation (A5) results in:

(A7) $$\sigma_{M}\left(\varepsilon \right)=\;e^{\frac{-K\varepsilon_{M}}{\frac{Cd\varepsilon_{C}}{dt}}}\left(e^{\frac{K\varepsilon_{M}}{\frac{Cd\varepsilon_{C}}{dt}}}-1\right)\frac{C}{100}=e^{\frac{-K\varepsilon }{C/100}}\left(e^{\frac{K\varepsilon }{C/100}}-1\right)\frac{C}{100}$$

Then the final expression of σ as a function of ε is expressed in terms of the parameters $K,k_{P},\;C,\;\gamma \;$ and n:

(A8) $$\sigma \left(\varepsilon \right)=\;e^{\frac{-K\varepsilon }{C/100}}\left(e^{\frac{K\varepsilon }{C/100}}-1\right)\frac{C}{100}+\;\varepsilon \left(k_{P}+\;\gamma {\left(1-e^{\varepsilon }\right)}^{n}\right)$$

In summary, we could fit these curves with the experimental data and extract the value of each parameter [1].
